# Catheter Ablation of Ventricular Premature Contractions Originating From RVOT With Interruption of the Inferior Vena Cava—A Case Report

**DOI:** 10.1111/anec.70034

**Published:** 2024-11-21

**Authors:** Liang Xiong, Jinzhu Hu, Dandan Wang, Juan Hua, Qi Chen

**Affiliations:** ^1^ Department of Cardiology The Second Affiliated Hospital of Nanchang University Nanchang China

**Keywords:** catheter ablation, electrophysiology, interruption of inferior vena cava, ventricular premature contractions

## Abstract

Ventricular premature contractions (VPC) originating from right ventricular outflow tract is the most common type of ventricular arrhythmias in clinic settings, which can be effectively cured by catheter ablation. Interruption of the inferior vena cava (IVC) is a rare vascular anomaly resulting from aberrant development during embryogenesis. Herein, we reported a very rare case of VPC originating from right ventricular outflow tract (RVOT) with interruption of IVC, which was successfully ablated by transfemoral vein approach.

## Graphical abstract

We reported a very rare case of ventricular premature contractions originating from right ventricular outflow tract with interruption of inferior vena cava, which was successfully ablated by transfemoral‐azygos vein approach.

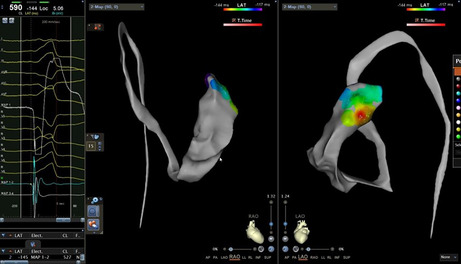

## 1 Case Presentation

**FIGURE 1:**
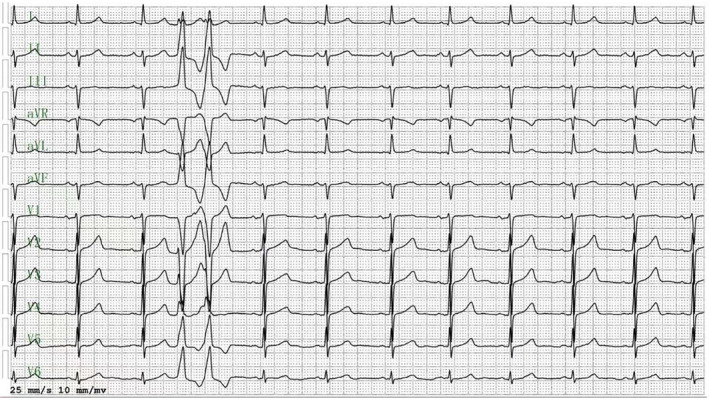
The 12‐lead electrocardiogram of ventricular premature contractions originating from the right ventricular outflow tract.

A 51‐year‐old female diagnosed as symptomatic ventricular premature contractions (VPC), the origin source of which was considered from right ventricular outflow tract according to the QRS complex characteristics of 12‐lead electrocardiogram (ECG), suffered from frequent occurrences of palpitation for 2 years. A 24‐h‐holter ECG revealed that the burden of VPC was up to 15% (20,123beats/day), which was characterized with left bundle branch block type, precordial transition in V4 (Figure 1). Giving fully consideration to the grievous symptom, the patient was admitted to hospital to receive catheter ablation therapy. Preoperative cardiac ultrasound examination showed no anatomic abnormalities, and blood routine and biochemical indexes were normal.

**FIGURE 2:**
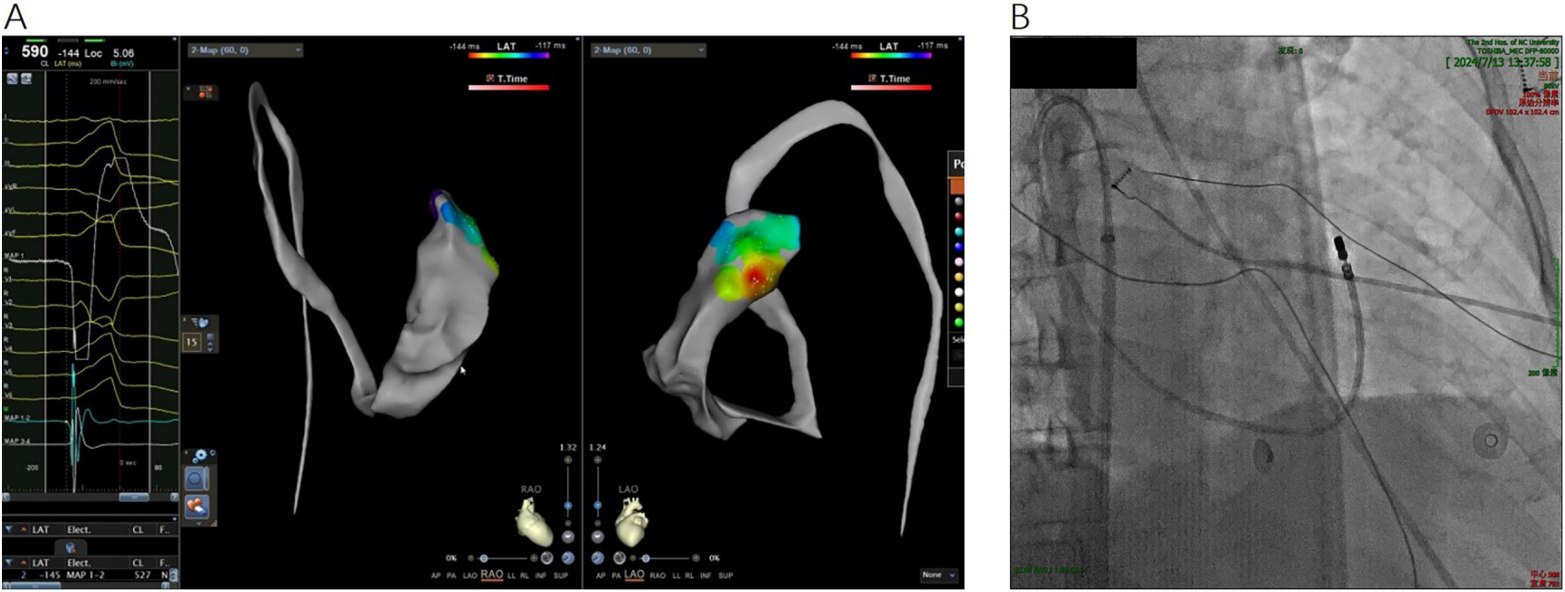
(A) The Carto system three‐dimensional anatomic model and activating mapping of ventricular premature contractions. (B) The X‐ray fluoroscopy image of RAO 30°. RAO, right anterior oblique.

During the operation, CARTO three‐dimensional mapping system (Biosense Webster, Diamond Bar, CA) was used for mapping and ablation. After intraoperative puncture of the femoral vein and placement of a 8.5F Swartz sheath, however, it was difficult to get the thermo cool (TC) ablation catheter (Biosense Webster) into the right ventricle guiding by right anterior oblique (RAO) 30° X‐ray fluoroscopy through the Swartz sheath. Then, Angiography of right atrium was immediately performed, showing that the inferior vena cava was interrupted and the blood of the lower part of body was refluxed into superior vena cava and right atrium through the azygos vein (Videos S1 and S2). However, we successfully completed this operation by the femoral vein approach. During the operation, the TC catheter was carefully sent upward along through the azygos vein, then entered the superior vena cava at the level of the aortic arch, and finally went down into right atrium. The catheter was meticulously bent and rotated to cross the tricuspid annulus into the RVOT. The Three‐dimensional anatomical model of partial RVOT was constructed and activated mapping of the VPC was performed carefully. The earliest activated site was located at anterior septum of RVOT, showing a near‐field presystolic potential preceding surface electrocardiogram QRS onset by more than 30 ms during VPC (Figure 2). The radiofrequency ablation parameters of TC catheter was set temperature at 43°C, power at 30 watts and brine speed at 17 ml per minute. The VPC was successfully abolished after 3 s of ablation energy delivery. Intravenous drip of isoproterenol was performed for stimulus of VPC recurrence and observation was made for 30 min, premature beat did not appear again. After 2 months of follow‐up, VPC did not appear again.

## 2 Discussion

Interruption of IVC are rare congenital abnormalities occurring in about 0.15% of the general population. In these variant patients of IVC interruption, the segment of the IVC between the hepatic and renal veins is almost absent, with blood from the liver typically draining directly into the RA through hepatic veins, and that from the lower extremities draining into the RA through the azygous system (Singh et al. 2011). In this case, we could clearly see that the diameter azygous vein cavity was extremely increased, through which the blood of the lower extremities drained into the right atrium.

There are alternative access strategies for catheter ablation of cardiac arrhythmias in the patients of interruption of IVC, including utilizing a superior venous approach via the jugular or subclavian veins and a transhepatic venous approach. According to a published study, the superior venous approach was successfully utilized to complete catheter ablation for patients with atrial fibrillation. However, the disadvantages of this approach were less favorable for transseptal puncture and stability of catheter manipulation (Kato et al. 2017). Another access is transhepatic venous approach, which is an inferior approach and more familiar to electrophysiologists for greater degrees of maneuverability for catheter manipulation and can serve for catheter ablation of a variety of cardiac arrhythmias, including atrioventricular nodal reentrant tachycardia, focal atrial tachycardia, cavotricuspid isthmus flutters, and atrial fibrillation. On the other hand, this approach also has several disadvantages including less compressible sites for hemostasis and more difficult for transseptal access (Nguyen et al. 2013).

We firstly reported that it might be feasible for catheter ablation of VPC originating from RVOT via femoral vein approach for patients with interruption of IVC. In this case, the TC catheter was carefully attempted to send upward through the azygos vein, then entered the superior vena cava at the level of the aortic arch, and finally went down into the right atrium. Fortunately, the catheter was accurately adjusted to cross the tricuspid annulus into the RVOT, which provided an important basis for successful ablation. The greatest significance of this case is to provide a new inspiration about the access selection for catheter ablation of VPC originating from RVOT for patients with interruption of the IVC.

## Author Contributions

Liang Xiong and Jinzhu Hu are in charge of surgery and article writing, Dandan Wang and Juan Hua are in charge of photo editing, and Qi Chen is in charge of article revision.

## Conflicts of Interest

The authors declare no conflicts of interest.

## Supporting information


**Video S1.** Angiography of right atrium of RAO 30°, showing that the inferior vena cava was interrupted and the blood of the lower part of body was refluxed into superior vena cava and right atrium through the azygos vein. RAO: right anterior oblique.

**Video S2.** Angiography of right atrium of LAO 45°, showing that the inferior vena cava was interrupted and the blood of the lower part of body was refluxed into superior vena cava and right atrium through the azygos vein. LAO: left anterior oblique.

## Data Availability

The data supporting the findings of this case report are available within the article. Additional data may be obtained from the corresponding author upon reasonable request.

## References

[anec70034-bib-0001] Kato, H. , S. Kubota , T. Goto , et al. 2017. “Transseptal Puncture and Catheter Ablation Via the Superior Vena Cava Approach for Persistent Atrial Fibrillation in a Patient With Polysplenia Syndrome and Interruption of the Inferior Vena Cava: Contact Force‐Guided Pulmonary Vein Isolation.” Europace 19, no. 7: 1227–1232. 10.1093/europace/euw095. 27174901

[anec70034-bib-0002] Nguyen, D. T. , R. Gupta , J. Kay , et al. 2013. “Percutaneous Transhepatic Access for Catheter Ablation of Cardiac Arrhythmias.” Europace 15, no. 4: 494–500. 10.1093/europace/eus315. 23385049

[anec70034-bib-0003] Singh, S. M. , P. Neuzil , J. Skoka , et al. 2011. “Percutaneous Transhepatic Venous Access for Catheter Ablation Procedures in Patients With Interruption of the Inferior Vena Cava.” Circulation. Arrhythmia and Electrophysiology 4, no. 2: 235–241. 10.1161/CIRCEP.110.960856. 21270102

